# Early Events in Actin Cytoskeleton Dynamics and E-Cadherin-Mediated Cell-Cell Adhesion during Epithelial-Mesenchymal Transition

**DOI:** 10.3390/cells9030578

**Published:** 2020-02-29

**Authors:** Irina Y. Zhitnyak, Svetlana N. Rubtsova, Nikita I. Litovka, Natalya A. Gloushankova

**Affiliations:** Institute of Carcinogenesis, N.N. Blokhin National Medical Research Center of Oncology, 115478 Moscow, Russia; irazhitnyak@gmail.com (I.Y.Z.); gaart2@gmail.com (S.N.R.); foxcovert9@gmail.com (N.I.L.)

**Keywords:** epithelial-mesenchymal transition, actin cytoskeleton, cell-cell adhesion, E-cadherin, EPLIN

## Abstract

Epithelial-mesenchymal transition (EMT) plays an important role in development and also in initiation of metastasis during cancer. Disruption of cell-cell contacts during EMT allowing cells to detach from and migrate away from their neighbors remains poorly understood. Using immunofluorescent staining and live-cell imaging, we analyzed early events during EMT induced by epidermal growth factor (EGF) in IAR-20 normal epithelial cells. Control cells demonstrated stable adherens junctions (AJs) and robust contact paralysis, whereas addition of EGF caused rapid dynamic changes at the cell-cell boundaries: fragmentation of the circumferential actin bundle, assembly of actin network in lamellipodia, and retrograde flow. Simultaneously, an actin-binding protein EPLIN was phosphorylated, which may have decreased the stability of the circumferential actin bundle. Addition of EGF caused gradual replacement of linear E-cadherin–based AJs with dynamic and unstable punctate AJs, which, unlike linear AJs, colocalized with the mechanosensitive protein zyxin, confirming generation of centripetal force at the sites of cell-cell contacts during EMT. Our data show that early EMT promotes heightened dynamics at the cell-cell boundaries—replacement of stable AJs and actin structures with dynamic ones—which results in overall weakening of cell-cell adhesion, thus priming the cells for front-rear polarization and eventual migration.

## 1. Introduction

Epithelial-mesenchymal transition (EMT) plays an important role in development and wound healing. Upon EMT, epithelial cells undergo profound changes in phenotype and behavior: they lose apical-basal polarization and stable cell-cell adhesion and acquire migratory activity by reorganizing their actin cytoskeleton to provide directional migration driven by dynamic protrusions at the leading edge [1,2]. EMT is highly implicated in pathological states such as fibrosis and cancer progression. The EMT program is regarded as the driver of invasion and metastatic dissemination of carcinoma cells [3,4]. It has long been thought that in cancer, EMT is a transcriptionally regulated program where tumor cells repress epithelial gene transcription and upregulate mesenchymal genes. It has been shown that E-cadherin, the main mediator of stable cell-cell adhesion in epithelial tissues, is transcriptionally repressed by EMT transcription factors (e.g., Snail, Twist, and Zeb). EMT transcription factors can also induce expression of mesenchymal markers such as N-cadherin, vimentin, and fibronectin [2,3,5].

In recent years, the traditional viewpoint of EMT as a transformation of epithelial cells into mesenchymal cells [6] was substantially revised. The in vitro and in vivo data demonstrated that in many cases, EMT drives cells from the epithelial state to a hybrid epithelial/mesenchymal phenotype (i.e., partial or intermediate EMT) [3,7,8,9,10]. Cells with the hybrid phenotype have both epithelial traits (E-cadherin-mediated cell-cell adhesion) and mesenchymal traits (migratory activity) that enable them to move collectively, as observed during embryonic morphogenesis, wound healing, cancer cell migration, invasion, and metastatic dissemination [11,12,13]. Hybrid phenotype of tumor cells and retention of E-cadherin allow them to join into clusters, which can enter blood circulation more effectively. Clusters of circulating tumor cells are more resistant to anoikis in the bloodstream and are more effective in extravasation and metastatic outgrowth in distant organs [14,15,16]. Cells in a hybrid epithelial/mesenchymal phenotype display plasticity. Intravital microscopy in mice showed that circulating tumor cells from a mammary carcinoma may spontaneously undergo EMT and then revert to epithelial phenotype [16]. Tumor cell phenotype is also modulated by signals from the tumor microenvironment. EMT can be triggered by TGF-β, EGF, HGF, and FGF produced by stromal cells [17,18,19,20]. HGF and TGF-β secretion by cancer-associated fibroblasts activated migration of carcinoma cells [21,22]. Highly dynamic EMT was described during tumor cell intravasation stimulated by tumor-associated macrophages that produced EGF and thereby activated migration of tumor cells [18,23].

Reorganization of the actin cytoskeleton is the obvious driving force behind the detachment of the cells and their directional migration, but the mechanisms controlling F-actin dynamics during EMT remain to be elucidated. Epithelial cell scattering is a well-known in vitro model for the study of EMT and it is considered that in the course of epithelial cell scattering, stimulation of lamellipodia formation and attachment at the free cell edges together with increased integrin-mediated tension perpendicular to the cell-cell boundaries leads to contraction of the rear and mechanical disruption of cell-cell contacts and promotion of directional migration [24]. However, this does not explain how in vivo, for example, in a tumor, cells can detach from one another in the absence of free edges. It may have been understandable in the context of down-regulation of E-cadherin expression, but in many cases E-cadherin expression may be retained during EMT in the cells of epithelial origin. Undoubtedly, the study of the alterations at the cell-cell interface is very important for understanding of weakening of cell-cell adhesion and induction of migratory characteristics during early stages of EMT.

In nontumorigenic epithelial cells and in carcinoma cells that preserve the circumferential actin bundle, E-cadherin-based AJs are organized linearly and associated with this bundle [25]. An actin-binding protein EPLIN (Epithelial Protein Lost in Neoplasm) additionally stabilizes the circumferential bundle by inhibiting actin depolymerization and cross-linking actin filaments [26]. Down-regulation of EPLIN isoforms’ expression was observed in many carcinoma cell lines and correlated with aggressiveness and negative clinical outcome of breast cancer [27,28].

In the present study, using various microscopic approaches we studied very early processes at the cell-cell interface during epidermal growth factor (EGF)-induced EMT. Our observations show that treatment with EGF of immortalized IAR-20 epithelial cells immediately triggers a series of events at the cell-cell boundaries that actively lead to increased dynamics of the actin cytoskeleton and adherens junctions (AJs). Within minutes of the stimulation, we observed local disruptions of the circumferential bundle, formation of actin-filled protrusions and retrograde flow at the cell-cell boundaries, phosphorylation of EPLIN and its disappearance from the circumferential bundle, and replacement of the linear AJs with dynamic punctate ones, while retaining E-cadherin expression. Punctate AJs colocalized with a mechanosensitive protein zyxin. Our findings imply that dynamic changes at the cell-cell boundaries, which result in overall weakening of cell-cell adhesion, are very early events of EMT, followed by front-rear polarization and migration of the cells.

## 2. Materials and Methods

### 2.1. Cell Culture and EGF Treatment

The IAR-20 line of immortal nontumorigenic epithelial cells was established from rat liver by Montesano et al. at the International Agency for Research on Cancer [29]. 1 × 10^5^ IAR-20 cells were seeded into 35-mm culture dishes in Dulbecco’s modified Eagle medium (DMEM) supplemented with 10% fetal bovine serum (FBS). 24 h after seeding, the medium was replaced with fresh DMEM containing 1% FBS for 16–20 h. Cells were treated with EGF (50 ng/mL) for different periods of time.

### 2.2. Antibodies and Reagents

The following primary antibodies were used for immunofluorescence microscopy: mouse monoclonal anti-E-cadherin, clone 36 (BD Transduction Laboratories); mouse monoclonal anti-β-actin, clone 4C2 (Abcam); mouse monoclonal anti-α-tubulin, clone DM1A (Sigma); mouse monoclonal anti-zyxin, clone 164D4 (Synaptic Systems); rabbit polyclonal anti-EPLIN (Novus Biologicals NB100-2305, lot A4); mouse monoclonal anti-β-catenin, clone 14 (BD Transduction Laboratories); rabbit polyclonal anti-p34-Arc/ARPC2 (Upstate, Merck), rabbit polyclonal anti-phospho-p44/42 MAPK (ERK1/2) (Cell Signaling Technology). The following secondary antibodies from Jackson ImmunoResearch were used: goat polyclonal anti-mouse IgG, IgG1, or IgG2a, or anti-rabbit IgG conjugated with AlexaFluor488, AlexaFluor594, or AlexaFluor647. AlexaFluor488-conjugated phalloidin (Molecular Probes) or TRITC-conjugated phalloidin (Fluka) were added to secondary antibodies. Horseradish peroxidase-conjugated goat polyclonal anti-mouse and anti-rabbit IgG antibodies (Jackson ImmunoResearch) were used for Western blot analysis. Other reagents were obtained from Sigma.

### 2.3. Immunofluorescence

For immunofluorescence, cells were seeded on glass coverslips in 35-mm culture dishes. For double staining for E-cadherin and β-actin or zyxin, cells were fixed with 1% paraformaldehyde (PFA) at room temperature for 10 min and permeabilized with cold methanol for 3 min. For double staining for actin and p34 or β-catenin, or triple staining for β-catenin/actin/EPLIN, cells were fixed with 3.7% PFA at room temperature for 15 min and permeabilized with 0.5% Triton X-100 for 5 min. Fixed specimens were incubated for 40 min with primary antibodies and subsequently, for 40 min with secondary antibodies. Mounted samples were examined with a Leica TCS SP5 confocal laser scanning microscope equipped with an HDX PL APO 63× objective or with a Nikon Eclipse Ti-E microscope equipped with a Plan Fluor 40× objective and ORCA-ER camera (Hamamatsu Photonics) controlled via NIS-Elements AR 3.22 software (Nikon).

### 2.4. Fluorescence Intensity

For making the fluorescence profiles, the Fiji/ImageJ software was used on double immunofluorescent staining images for F-actin/β-catenin or p34/E-cadherin. For making an individual plot, a 20- or 10-µm long straight-line selection was drawn perpendicular to an AJ. The option ‘List’ in the Plot window returned the intensity value for every point on the line selection. For p34/E-cadherin images, the selections were not symmetrical: the starting points of the selections were located on the AJ side of a cell-cell boundary and the selections were extended to the lamella side. Intensities of 30 plots of 30 different cell-cell contact zones for every time-point of EGF treatment were averaged, and the graphs were built using MS Excel (Microsoft, Redmond, WA, USA) and GraphPad Prism 6 (GraphPad Software, San Diego, CA, USA). Data are expressed as mean ± SEM.

For EPLIN image analysis, the Fiji/ImageJ software was also used. Freehand selection tool was used to select the area of an AJ and background area near the contact zone on the E-cadherin confocal images, and then measurements were acquired on the corresponding EPLIN images. Fluorescence intensity (FI) was calculated as FI = (MGV(EPLIN) − MGV(BG))/MGV(BG), where “MGV” is mean gray value and “BG” is background. The graphs were built using GraphPad Prism 6. Data are expressed as mean ± SEM, N = 35.

### 2.5. Constructs and Transfection

The plasmid expressing GFP-E-cadherin was kindly provided by S.M. Troyanovsky (Northwestern University, Chicago, IL, USA) [30]. GFP-tagged regulatory light chain (RLC) of nonmuscle myosin IIA was a gift from Rex L. Chisholm (Center for Genetic Medicine, Feinberg School of Medicine, Northwestern University, Evanston, IL, USA) [31]. The actin marker F-tractin (ITPKA-9–40) tagged with tdTomato was a gift from M. Schell (Uniformed Services University, Bethesda, MD, USA) [32]. The mKate2-zyxin construct was purchased from Evrogen (Russia). All transfections were carried out using Lipofectamine® LTX and PLUS™ transfection reagents (Invitrogen, ThermoFisher Scientific) according to the One Tube Protocol by Invitrogen. Clones were obtained after 2 wk of selection with G418 (1mg/mL).

### 2.6. Live-Cell Imaging, Tracking, and Kymograph Analysis

For live-cell differential interference contrast (DIC) imaging, cells were seeded into 35-mm glass bottom culture dishes (MatTek Corporation). 20 min before imaging, the medium was exchanged for phenol-red-free DMEM/F-12 medium with L-glutamine and HEPES supplemented with 1% FCS. Cells were observed with a Nikon Eclipse Ti-E microscope (Plan Fluor 40× objective; ORCA-ER camera by Hamamatsu Photonics (Hamamatsu City, Japan); NIS-Elements AR 3.22 software (Nikon, Tokyo, Japan)). After 1 h of control imaging, EGF was added.

For cell tracking, the position of the cell’s center of mass throughout a 6-h time-lapse DIC sequence (1 frame/5 min) was determined. The coordinates were then plotted starting from the zero point onto graphs using MS Excel.

For kymograph analysis, DIC sequences were shot with a Nikon Eclipse Ti-E microscope (Plan Fluor 100× objective) at 1 frame/3 s for 30 min before the addition of EGF and for 30 min after the addition of EGF. Kymographs were produced using the Fiji/ImageJ software with the Multiple Kymograph plugin. A Straight-line selection was drawn perpendicular to the cell edge. The lengths of lamella overlaps at selected time points were measured. Data are expressed as mean ± SEM, N = 20. Mann-Whitney U Test was used for statistical analysis.

For the myosin retrograde flow, the cells were imaged with the ORCA Flash 4.0V3 camera (Hamamatsu Photonics) at 1 frame/min. A region of interest was cropped out and subjected to deconvolution with Huygens Essential (SVI Imaging, The Netherlands). The Microscopic template was extracted from images, and a built-in Deconvolution template (Confocal_Low_Signal) was used with a theoretical PSF. The deconvolved frame sequence was analyzed using the quantitative fluorescent speckle microscopy tool [33].

To study the motility of beads on the cell surface, 0.5 µm polystyrene beads (Polysciences, Warrington, PA, USA) were coated with Concanavalin A (ConA beads) and resuspended in DMEM just before use. Culture medium in 35-mm glass bottom culture dishes was replaced with the medium containing ConA beads. Individual beads spontaneously attached to the cell surface. Time-lapse sequences were shot at 1 frame/min. Bead translocations were tracked using Fiji/ImageJ.

Cells doubly transfected with GFP-E-cadherin and mKate2-zyxin were imaged with the Leica TCS SP5 confocal laser scanning microscope equipped with an HDX PL APO 63x objective at 1 frame/2 min.

### 2.7. Western Blot Analysis

Cells were washed twice with Wash Buffer (10 mM Tris-HCl pH7.5, 0.5 mM EDTA, 150 mM NaCl) and lysed with Lysis Buffer (Wash Buffer including 0.5% Na deoxycholate, 1% NP-40, protease inhibitor cocktail (Roche), and phosphatase inhibitor cocktail (Sigma)). Samples were mixed with 5x Sample Buffer (250 mM Tris-HCl pH6.8, 10% SDS, 30% Glycerol, 5% β-mercaptoethanol, 0.02% bromophenol blue), heated for 10 min at 95 °C, and loaded onto SDS-polyacrylamide gel in equal protein concentrations according to the SDS-PAGE Bio-Rad protocol. Resolved proteins were transferred to Amersham HybondTM-P PVDF membranes (GE Healthcare, Chicago, IL, USA). Membranes were blocked with 5% m/v bovine serum albumin solution (AppliChem, Darmstadt, Germany) in Tris-buffered saline with 0.1% *v*/*v* of Tween 20 (AppliChem) for 1 h followed by incubation with the primary antibodies at 4 °C overnight. After washing, peroxidase-conjugated secondary antibodies were applied for 1 h at room temperature. Blotted protein bands were detected using Pierce ECL Western Blotting Substrate (ThermoFisher Scientific, Waltham, MA, USA), and chemiluminescence images were captured by Image Quant LAS4000 (GE Healthcare).

## 3. Results

### 3.1. EGF-Induced Cell Scattering

**Figure 1:**
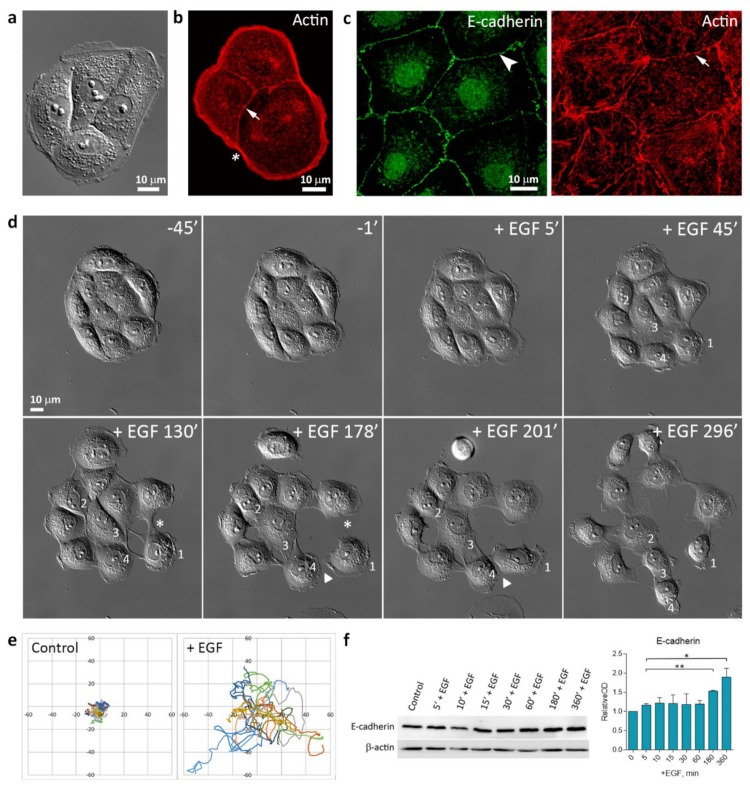
IAR-20 epithelial cells undergoing epidermal growth factor (EGF)-induced epithelial-mesenchymal transition (EMT). (**a**) In sparse culture, control IAR-20 epithelial cells form islands. DIC-microscopy. (**b**) In IAR-20 cells, the actin cytoskeleton is organized into the marginal actin bundle (asterisk) and circumferential actin bundles (arrow). (**c**) E-cadherin-based AJs (arrowhead) in an IAR-20 monolayer exhibit linear organization and colocalize with circumferential actin bundles (arrow). (**d**) Scattering of IAR-20 epithelial cells in response to EGF (50 ng/mL). In the control (45 min and 1 min before treatment with EGF), cells are joined into an island with stable cell-cell contacts. Addition of EGF leads to stimulation of protrusive activity at the free cell edges (cell 1), disruption of cell-cell contacts (asterisks), and initiation of cell migration. The migratory cells can form new transient contacts with neighboring cells (arrowheads). Both individual (cell 1) and collective (cells 2, 3, and 4) migration can be observed. Selected frames from Supplementary Video S1. (**e**) The centroid trajectories of cells migrating for 6 h. (**f**) Western blot showing the expression levels of E-cadherin in IAR-20 cells treated with EGF. β-actin was used as loading control. Densitometry results are averaged across three independent experiments. Data are presented as mean ± SEM, * *p* < 0.05, ** *p* < 0.002.

In sparse culture, normal rat liver IAR-20 epithelial cells formed islands, which merged into a monolayer as the culture grew denser. As revealed by immunofluorescent staining, individual cells and cells joined into islands had a marginal actin bundle at the free edges and circumferential bundles which colocalized with linear AJs. (Figure 1a–c).

The linear E-cadherin-based AJs were stable and dissolved only during mitosis. Treatment with EGF resulted in morphological changes in IAR-20 cells and cell scattering. In islands, within mere minutes of stimulation, we observed induction of protrusive activity at the free cell edges, disruption of cell-cell contacts, and initiation of cell migration. Time-lapse imaging showed that EGF treatment induced random cell migration, cells could move individually, establish transient contacts with other cells, or migrate as a group. (Figure 1d,e and Video S1). Western blot analysis showed that at least 6 h after the addition of EGF, when cells disrupt cell-cell contacts and migrate on substrate, E-cadherin expression was maintained. After 3 h of EGF treatment, we observed an increase in E-cadherin levels. (Figure 1f).

### 3.2. EGF-Stimulated Protrusive Activity in the Zones of Cell-Cell Contacts

**Figure 2:**
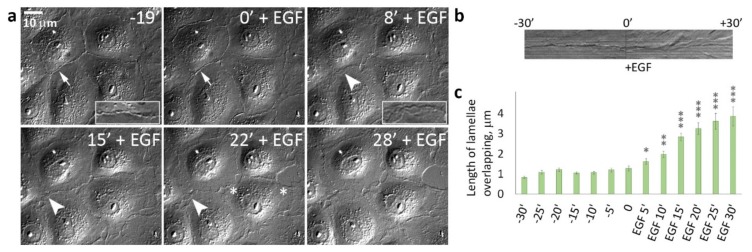
Disappearance of contact paralysis in IAR-20 epithelial cells after the addition of EGF. (**a**) Selected frames from Video S2. The cell-cell boundaries (indicated with arrows and arrowheads) are enlarged in boxed regions. In control cells, the thin “scars” of cell-cell contacts are seen at the cell-cell boundaries (arrows). In the presence of EGF, cell-cell interfaces became highly unstable (arrowheads) and overlapping lamellae were seen at the cell-cell boundaries. Asterisks indicate sites of disruption of cell-cell contacts. (**b**) Kymograph generated at the site of the cell-cell contact shows contact paralysis before EGF addition and appearance of protrusive activity caused by addition of EGF. (**c**) Length of lamellae overlapping at the cell-cell boundaries. * *p* < 0.05, ** *p* < 0.001, *** *p* < 0.0001.

Earlier, in MDCK culture treated with HGF, it was shown that cell scattering was due to stimulation of protrusive activity at the free cell edges, attachment of protrusions and integrin-dependent actomyosin contractility that transmitted to the rear cell-cell boundaries, and passive disruption of cell-cell contacts [24]. As cells surrounded in tissues by neighboring cells do not have free edges, we decided to investigate whether cells in dense cultures are capable of disrupting cell-cell adhesion in the presence of EGF. DIC live-cell imaging showed that in dense cultures, control IAR-20 epithelial cells did not form pseudopodia in the zones of cell-cell contacts demonstrating robust contact paralysis. In contrast, addition of EGF resulted in dramatic changes at the cell-cell boundaries: within only 5–10 min of EGF treatment protrusions developed all around cell-cell boundaries and cell-cell boundaries became highly unstable. Instead of the thin “scar” of a stable cell-cell contact, lamella overlaps between contacting cells were observed (Figure 2 and Video S2). Disappearance of contact paralysis was the first sign of EGF-induced EMT.

### 3.3. Reorganization of the Actin Cytoskeleton and AJs during EGF-Induced EMT

**Figure 3:**
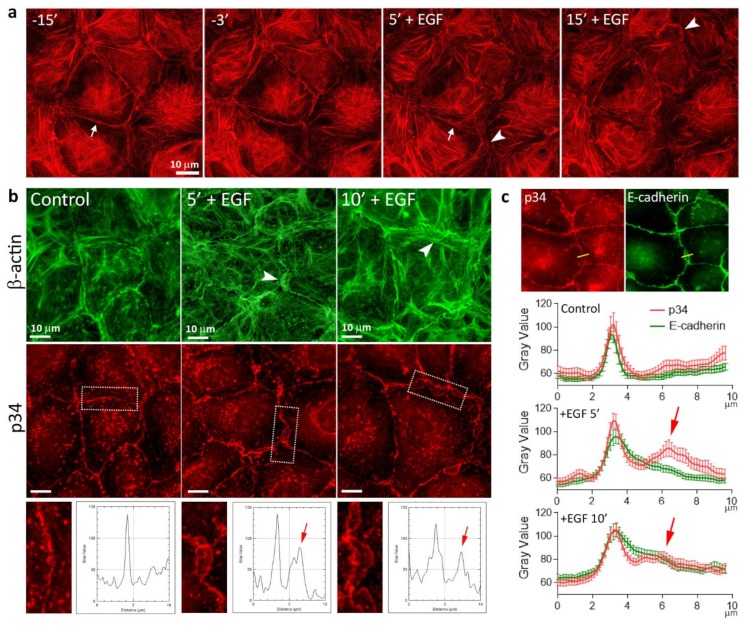
Reorganization of the actin cytoskeleton in IAR-20 cells treated with EGF. (**a**) Disruption of the circumferential actin bundle (arrow) and appearance of pseudopodia at the cell-cell-boundaries (arrowheads) after the addition of EGF in IAR-20 cells stably expressing F-tractin-tdTomato. Selected frames from Supplementary Video S3. (**b**) Immunostaining of p34 and β-actin. In control cells, the Arp2/3 complex resides at cell-cell boundaries. In EGF-treated cells, Arp2/3 is enriched in lamellipodia close to cell-cell boundaries (arrowheads) where the disintegrated circumferential bundle is also seen. Dashed rectangles indicate the cell-cell boundaries enlarged in boxed regions with corresponding fluorescence intensity profiles. Arrows indicate p34 fluorescence intensity peak corresponding to the pseudopodia edge. (**c**) Immunostaining of p34 and E-cadherin (Figure S1) and corresponding fluorescence intensity profiles. In control cells, p34 colocalizes with E-cadherin. In EGF-treated cells, a second, smaller peak of p34 intensity appears which corresponds to the extending edges of the pseudopodia (arrows).

As changes in morphology and cell-cell adhesion in the cells undergoing EMT are undoubtedly coordinated with the actin cytoskeleton reorganization, to investigate EGF-induced cytoskeletal rearrangements in living cells, we established a line of IAR-20 cells stably expressing the F-actin marker F-tractin-tdTomato, which does not influence actin dynamics or function [32]. Using confocal live-cell imaging, we observed rapid destruction of circumferential actin bundles and appearance of pseudopodia at the cell-cell-boundaries within 5–10 min of EGF treatment (Figure 3a and Video S3). Extension of the pseudopodia depended on assembly of actin in Arp2/3-branched actin networks. Using immunofluorescent staining for the p34 (ARPC2) subunit of the Arp2/3 complex as a marker of growing branched actin networks, we found that in control cells, at cell-cell boundaries p34 colocalizes with E-cadherin. By 5–10 min after the addition of EGF, Arp2/3 also appeared at the extending edge of pseudopodia, which appeared close to the cell-cell boundaries (Figure 3b,c and Figure S1).

**Figure 4:**
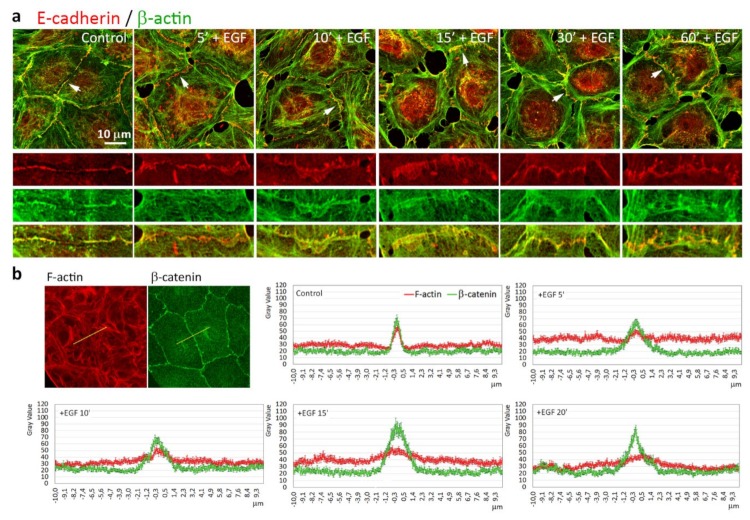
Rearrangement of E-cadherin-based AJs in parallel with reorganization of the actin cytoskeleton in IAR-20 cells treated with EGF. (**a**) Immunostaining of E-cadherin and β-actin. Top—red and green channels merged. The cell-cell boundaries enlarged below are indicated with arrows. Disorganization of AJs begins 5 min after addition of EGF, simultaneously with dissolution of the circumferential actin bundle and formation of lamellipodia. Beginning from 10–15 min after the addition of EGF, punctate AJs were connected with straight actin fibers appear. (**b**) Fluorescence intensity profiles of F-actin and β-catenin in the zones of cell-cell contacts. N = 30 for every graph.

We used immunofluorescence microscopy to compare, in parallel, the actin cytoskeleton and AJs in IAR-20 cells before and at different times after the addition of EGF. In control cells, E-cadherin accumulated in linear AJs that colocalized with circumferential actin bundles (Figure 4a). Addition of EGF resulted in rapid reorganization of the actin cytoskeleton in the contact zones. After 5 min of incubation with EGF, circumferential actin bundles were already fragmented, lamellipodia filled with actin filaments appeared at the cell-cell boundaries, and simultaneously, linear AJs became discontinuous. Beginning from 10–15 min after the addition of EGF, punctate AJs appeared at the cell-cell boundaries and actin accumulated in these AJs. Longer, more prominent AJs were associated with straight actin fibers.

Fluorescence intensity profiles of filamentous actin and β-catenin in the zones of cell-cell interaction showed that while in control cells, F-actin and β-catenin accumulated in the thin (about 1 µm wide) zone of the AJs; in EGF-treated cells, the zone of the AJs expanded up to 3–5 µm. F-actin in the zone of cell-cell interaction did not form the sharp intensity peak as in control cells (Figure 4b). Thus, our data demonstrated that addition of EGF resulted in rapid rearrangement of E-cadherin-based AJs and the actin cytoskeleton at the cell-cell boundaries: disappearance of linear AJs and circumferential actin bundle, and formation of lamellipodia and punctate AJs associated with straight actin fibers.

### 3.4. Retrograde Flow in the Cells Undergoing EMT

The continuous centripetal movement (retrograde flow) of peripheral F-actin has been observed in many types of cells (e.g., fibroblasts, keratocytes, leukocytes) [34,35,36]. Recently, it was found that stacks of myosin filaments also flowed centripetally [37]. External markers such as Concanavalin A-coated latex beads (ConA beads), placed onto the dorsal surface of the cells on the free edges—attaching to the actin cytoskeleton via surface glycoproteins—also migrated centripetally over the cell surface [38]. The rearward actin flow and the rearward flow of ConA beads is driven by a myosin IIA-dependent centripetal force [39,40].

**Figure 5:**
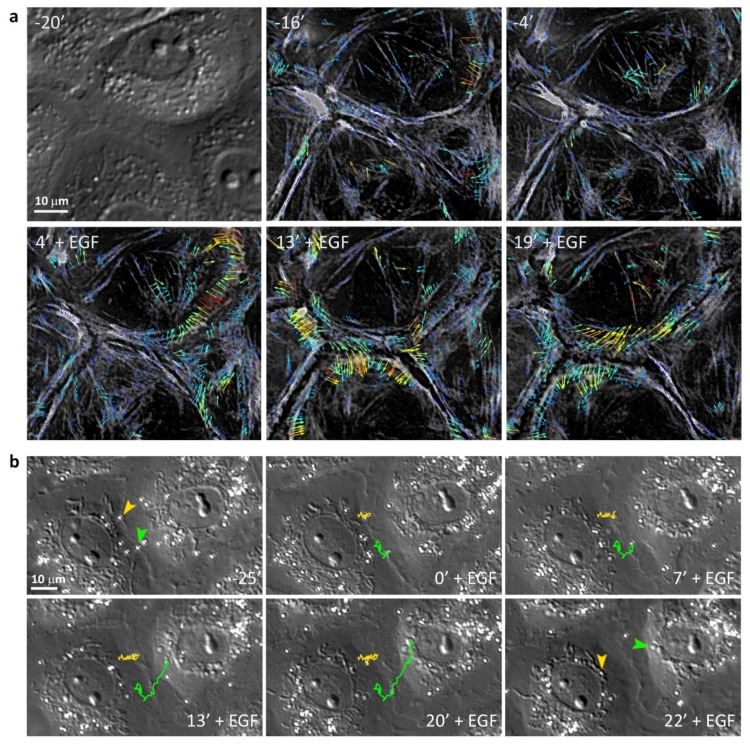
Retrograde flow in IAR-20 cells caused by addition of EGF. (**a**) Myosin retrograde flow in GFP-RLC expressing IAR-20 cells treated with EGF. Selected frames from Video S4. “-20’”–a corresponding DIC image to illustrate the integrity of the control monolayer. Myosin “speckles” were tracked using qFSM plugin for MatLab. In control cells, the slow speckle movement is only observed along the AJs. Immediately after EGF addition, the speckles begin to move centripetally, at much faster speeds. (**b**) Tracks of migrating ConA beads attached to the dorsal cell surface in the zone of cell-cell contact before and after the addition of EGF. Selected frames from Video S5. Arrowheads indicate initial and final positions of the beads.

To study the dynamics of nonmuscle myosin II in living cells, IAR-20 cells were transfected with a construct of GFP-tagged regulatory light chain of nonmuscle myosin IIA (GFP-RLC) [31]. We collected time-lapse images of GFP-RLC expressing IAR-20 cells and analyzed the movement of myosin speckles using fluorescent speckle microscopy approach. In control cells, we observed retrograde flow of speckles only at the free edges of the cells, but in dense cultures, at the cell-cell boundaries the speckles moved slowly along the cell-cell contacts. Immediately after the addition of EGF, a much faster centripetal flow of speckles appeared at the cell-cell boundaries (Figure 5a and Video S4).

We also studied the retrograde flow by analyzing the positions of ConA beads (d = 0.5 µm) added to the culture medium which spontaneously attached to the dorsal surface of the cells. The beads that attached to the cell surface near cell-cell contacts did not move centripetally but only moved very slowly along the cell-cell border. After the addition of EGF, simultaneously with formation of pseudopodia, the beads started migrating centripetally at an average rate of about 0.7 µm/min (Figure 5b and Video S5). These data show that addition of EGF immediately induces retrograde flow and generation of centripetal force in IAR-20 epithelial cells.

### 3.5. Highly Dynamic AJs in the Cells Undergoing EMT

**Figure 6:**
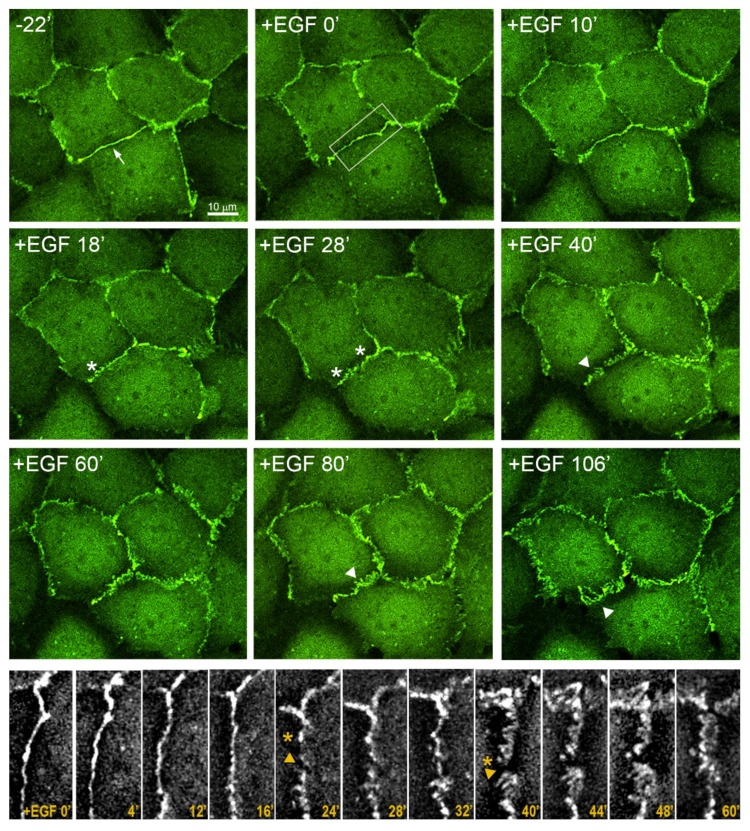
Rearrangement of AJs in EGF-treated IAR-20 cells. IAR-20 cells were stably transfected with GFP-E-cadherin. Top—in the presence of EGF, the linear AJs are replaced with punctate AJs. The arrow indicates linear AJs, asterisks indicate dotlike clusters, and arrowheads indicate punctate AJs. The boxed area denotes the cell-cell boundary enlarged in the bottom part. Selected frames from Video S6. Bottom—in EGF-treated cells, AJs are very dynamic. AJs can grow, rearrange, and change their position. Arrowheads indicate disassembly of AJs; asterisks indicate newly formed dotlike AJs. Selected frames from Video S7. A corresponding DIC image demonstrating the integrity of the monolayer is shown in Figure S2.

Epithelial cells have very stable linear AJs. To analyze possible changes in E-cadherin-based AJs as a result of EGF-stimulated EMT, IAR-20 cells were stably transfected with a GFP-E-cadherin construct which did not affect the distribution of endogenous E-cadherin and colocalized with it in AJs [30]. Using confocal live-cell imaging, we observed the distribution of AJs in the cells for 2–3 h. In normal epithelial cells before treatment with EGF, AJs were present as linear assemblies along the cell-cell boundaries. In the course of EGF-induced EMT, remodeling of E-cadherin-based AJs was observed (Figure 6 and Videos S6 and S7). Due to additional exogenous E-cadherin expression, the dynamics of EGF-induced changes in this cell line was slightly slower. By 15–20 min after the addition of EGF, linear AJs disappeared and dotlike clusters appeared at the cell-cell boundaries. Later (after 40 min of incubation with EGF), E-cadherin-based AJs manifested as dotlike adhesions or strands arranged mostly perpendicular to the cell-cell boundaries. Punctate E-cadherin-based AJs in the cells treated with EGF were very dynamic—able to grow, rearrange, and change their position in the zone of contact or disappear altogether.

**Figure 7:**
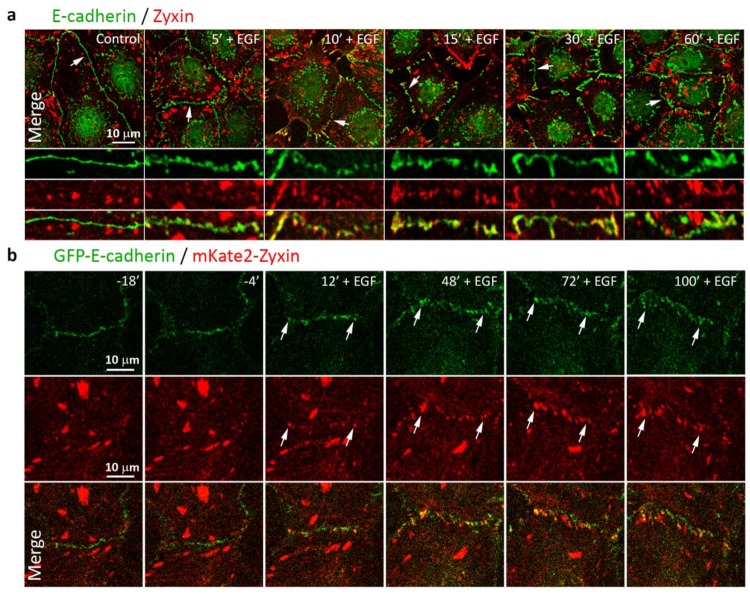
Accumulation of zyxin in AJs of IAR-20 cells treated with EGF. (**a**) Immunostaining of E-cadherin and zyxin. In control cells, zyxin does not accumulate in linear AJs. In EGF-treated cells, zyxin accumulates in punctate AJs. (**b**) IAR-20 cells stably expressing GFP-E-cadherin were transfected with mKate2-zyxin. Arrows indicate colocalization of zyxin with punctate AJs. Selected frames from Video S8.

We assessed the distribution of a mechanosensitive protein zyxin in the AJs in control and EGF-treated IAR-20 cells. Zyxin had been shown to be essential for repair of actin bundles and reorganization of actin cytoskeleton [41,42]. In various cells, zyxin is localized prominently in focal adhesions, though it has been shown that in Human Umbilical Vein Endothelial Cells, zyxin was also concentrated in punctate AJs in a tension-dependent manner [43]. Using immunofluorescent staining, we revealed prominent differences in the accumulation of zyxin in AJs of the IAR-20 cells before and after treatment with EGF. In control cells, zyxin colocalized with focal adhesions and was not present in stable linear AJs. After treatment with EGF for 5–10 min, zyxin began to colocalize with dotlike clusters of E-cadherin. Later, zyxin continued to accumulate in punctate AJs (Figure 7a). Next, we transfected IAR-20 cells stably expressing GFP-E-cadherin with mKate2-zyxin and observed the distribution of E-cadherin and zyxin by confocal live-cell imaging. This analysis supported the data of immunostaining. Zyxin was not revealed in the zones of linear AJs in the control cells, but at 10–12 min after the addition of EGF it began to appear in the zones of AJ remodeling (Figure 7b and Video S8). The appearance of zyxin indicates activation of actomyosin contractility at the sites of cell-cell adhesion during EMT.

### 3.6. EGF-Induced Phosphorylation of EPLIN

**Figure 8:**
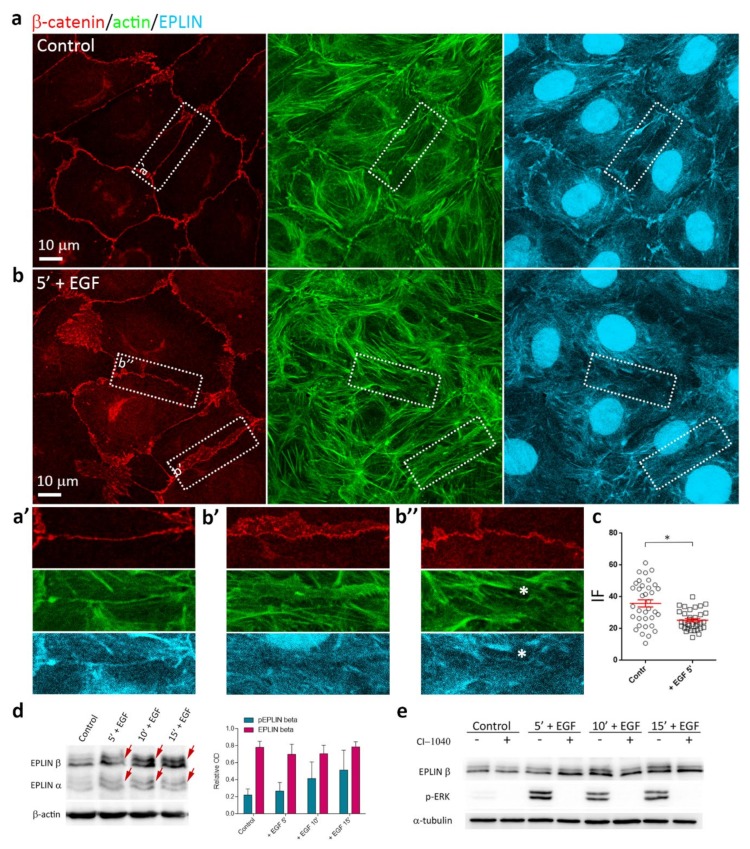
Effects of EGF on EPLIN in IAR-20 cells. (**a**), (a’) In control IAR-20 cells, EPLIN colocalizes with the circumferential actin bundles at cell-cell boundaries. (**b**) Addition of EGF leads to release of EPLIN from the zones of disorganization or disappearance of the circumferential bundles (Figure 8b’,b’’). EPLIN colocalizes with the remaining intact circumferential bundle (Figure 8b’’, asterisk). (**c**) EPLIN fluorescence intensity at the cell-cell boundaries in control and EGF-treated cells. Circles and squares represent individual cells, N = 35, * *p* < 0.001. (**d**) Western blot analysis of EPLIN phosphorylation (5% PAAG). Arrows indicate up-shifted bands of phosphorylated EPLIN in the cells treated with EGF. β-actin was used as loading control. Densitometry results are averaged across three independent experiments. Data are presented as mean ± SEM. (**e**) MEK inhibitor CI-1040 (4 µM), which inhibits phosphorylation of ERK (p-ERK), significantly decreases the levels of phosphorylated EPLIN at 10 min and 15 min after the addition of EGF. α-tubulin was used as a loading control.

The actin-binding protein EPLIN is known to stabilize the circumferential actin bundles. Disappearance of EPLIN isoforms in cancer cells sheds light on the important role of EPLIN in the maintenance of cohesion of nontumorigenic epithelial cells but not of migrating tumor cells [26,27,28]. As it has been shown that transfection of siRNA to EPLIN resulted in disruption of cell-cell adhesion, converting linear AJs into punctate AJs associated with radial actin bundles [44], we decided to investigate the localization of EPLIN in IAR-20 cells before and after the addition of EGF. Immunofluorescence microscopy showed association of EPLIN with circumferential actin bundles in control IAR-20 cells (Figure 8a,a’). After 5 min of incubation with EGF, in the regions of disorganized circumferential actin bundles, EPLIN staining was no longer observed; however, EPLIN colocalization with the remaining AJs and circumferential actin bundles persisted (Figure 8b,b’,b’’). In cells treated with EGF for 5 min, we observed a statistically significant decrease of EPLIN fluorescence intensity in the AJ regions compared to control cells (Figure 8c). In cells treated with EGF for longer, EPLIN was observed in straight actin fibers associated with punctate AJs (Figure S3).

Using Western blotting, we found that in IAR-20 cells, addition of EGF caused prominent phosphorylation of EPLIN. Within minutes of stimulation by EGF phosphorylated forms of EPLIN-α and EPLIN-β, isoforms were detected as up-shifted bands (Figure 8d) while total EPLIN levels did not change (Figure S4). As EPLIN is a well-known target of extracellular signal-regulated kinase (ERK) [45], we used a specific MEK1/2 inhibitor CI-1040 to confirm that the electrophoretic mobility shift of EPLIN in EGF-treated IAR-20 cells was due to phosphorylation. Pretreatment with CI-1040 inhibited EGF-induced phosphorylation of EPLIN (Figure 8e). Thus, EGF may activate rapid phosphorylation of EPLIN that is associated with the disruption of circumferential actin bundles.

## 4. Discussion

EMT has been traditionally viewed as a switch from expression of epithelial markers to mesenchymal ones, such as loss of E-cadherin and occludin and appearance of N-cadherin and vimentin in epithelial cells. Transcriptional inhibition of expression of the main molecule of epithelial cell-cell adhesion—E-cadherin—has long been considered the key feature of EMT. However, growing evidence indicates that EMT is not a binary switch between epithelial and mesenchymal states, but that cells can exhibit other modes of EMT, retaining epithelial markers and acquiring mesenchymal properties—as recent work using intravital microscopy in mice demonstrated, cells from a mammary carcinoma pool may spontaneously undergo EMT and acquire migratory activity but revert to epithelial phenotype in growing metastases [16]. EMT may be a highly dynamic process, such as the induction of motility of tumor cells in association with macrophages consistent with their paracrine interaction that was described in PyMT tumors [18]. The key events of dynamic EMT are loss of stable cell-cell adhesion and acquisition of the migratory phenotype by epithelial cells. It is considered that scattering of epithelial cells from islands in sparse cultures during EMT is caused by activation of pseudopodia formation on the free cell edges and the traction forces the pseudopodia exert on the cell body after their attachment to the substrate with integrin-based focal adhesions. This would lead to mechanical detachment of the cells from one another and passive disruption of cell-cell junctions as a result of increased actomyosin contractility [24]. However, changes at the cell-cell boundaries leading to disruption of the cell-cell contacts and allowing cells to detach from and migrate away from their neighbors have not been studied in sufficient detail.

**Figure 9:**
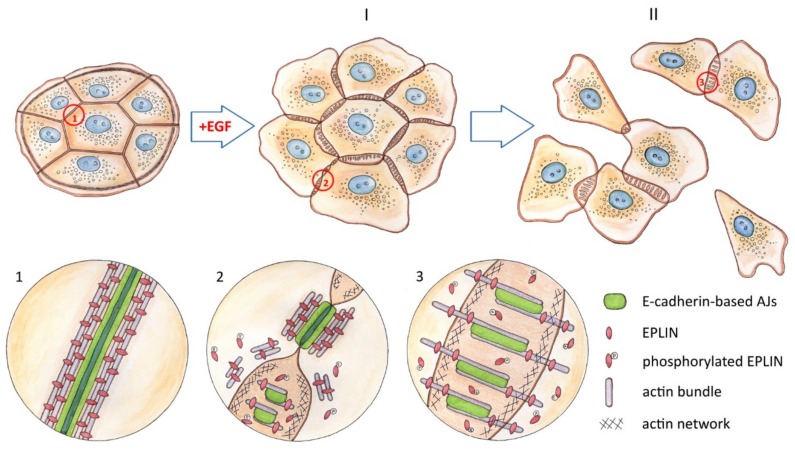
Schematic representation of the actin cytoskeleton reorganization and rearrangement of E-cadherin-based AJs during EGF-induced EMT (created by I.Y. Zhitnyak).

In our work, we used dense rather than sparse cultures of IAR-20 cells, where the cells did not have free edges, and treated them with EGF for varying amounts of time. Using this approach, we were the first to observe very early dynamic changes at cell-cell boundaries that can possibly promote weakening of cell-cell adhesion and subsequent acquisition of the motile phenotype by the epithelial cells. In IAR-20 cells, within 5 min of EGF treatment, we noticed disappearance of the contact paralysis and appearance of pseudopodia at the cell-cell boundaries (Figure 9). A little later—after 15–20 min of EGF treatment—gaps appeared at the cell-cell borders and the cells began to detach from one another. During the same early stages of treatment, dramatic reorganization of the actin cytoskeleton at the cell-cell boundaries was taking place. At 3–5 min after the addition of EGF, we observed fragmentation of circumferential actin bundles associated with linear AJs and appearance of pseudopodia close to AJs as a result of Arp2/3-mediated assembly of branched actin network. By 15 min of EGF treatment, straight actin fibers began to grow from nascent actin structures associated with newly formed punctate AJs.

Simultaneously with the induction of pseudopodial activity after the addition of EGF, we observed appearance of retrograde flow at the cell-cell boundaries which was revealed by myosin speckle displacement and by the centripetal movement of latex beads across the dorsal surface of the cells. The flow leads to formation of contractile actomyosin structures that create centripetal force. Appearance of retrograde flow in the zone of previous robust contact paralysis during the early stages of EMT is evidence of activation of intracellular processes in the vicinity of a cell-cell contact which lead to acquisition of migratory properties by the cells. In a recent work by Vassilev et al. [46], it was shown that retrograde flow at the leading edge of a cell plays an important role in front-rear polarization and directional migration of neural crest and glioblastoma cells. According to the authors’ hypothesis, retrograde flow of vesicles carrying α-catenin and 115RhoGEF causes concentration of 115RhoGEF in the perinuclear region, where it activates Rho. Rho activation triggers contraction of the rear which leads to directional migration. We propose that the appearance of retrograde flow at the early stages of EMT may also lead to centripetal displacement of α-catenin released in the process of linear AJ destruction and, in turn, to induction of front-rear polarization and directional cell migration. Further, more-detailed studies will be required to confirm this hypothesis.

Simultaneously, the continuous linear E-cadherin-based AJs were being disrupted. Importantly, in the IAR-20 cells undergoing EGF-induced EMT, E-cadherin-based AJs did not disappear completely: stable linear AJs were replaced with dynamic punctate AJs. In contrast with the linear AJs in control epithelial cells, these punctate AJs were extremely dynamic and unstable, undergoing continuous remodeling. The initial dotlike clusters of E-cadherin grew into punctate AJs associated with straight actin fibers as a result of actomyosin contractility. A mechanosensitive protein, zyxin, accumulated in these punctate AJs but not in the stable linear AJs. Earlier, we found similar punctate AJs in cells transformed in vitro by carcinogens or the Ras oncogene and demonstrated that these AJs were extremely dynamic and therefore incapable of supporting stable cell-cell adhesion [47]. E-cadherin-based AJs are essential for collective migration and collective invasion of carcinoma cells [13,48,49]. In our previous works, we showed that dynamic AJs were important for collective migration of transformed epithelial cells and could be involved in adhesion of neoplastic cells to normal epithelial cells and invasion of epithelial structures [50,51].

We propose that AJ reorganization and weakening of cell-cell adhesion in IAR-20 cells undergoing EMT are due to disruption of the circumferential actin bundles. EPLIN stabilizes the circumferential actin bundles that are connected with AJs [44]. We detected increased phosphorylation of EPLIN within minutes of the addition of EGF. Earlier, it has been demonstrated that phosphorylation of EPLIN by ERK reduces its affinity for F-actin. Phosphorylation of EPLIN during EGF treatment leads to its degradation through ubiquitin-proteasome-dependent mechanism [52]. We propose that this results in a decreased ability of EPLIN to stabilize the circumferential actin bundle, which weakens the bundle structure and leads to release of monomeric actin. In a cell, there is an intense competition between actin-polymerizing systems for the limited pool of the globular actin [53,54]. EGFR signaling has also been shown to activate Rac1 through different guanine nucleotide exchange factors (GEFs) [55,56,57]. We think that, as a result, Rac1-WAVE2-Arp2/3 axis signaling and concurrent disruption of the circumferential actin bundle caused by EGF may lead to fast polymerization of an actin network and pseudopodia formation at the cell-cell boundaries.

Thus, our data show that, contrary to previous findings, early EMT not only works visibly on the free cell edges, but results in dynamic changes at the cell-cell boundaries as well. Even at its very beginning, stable epithelial cell-cell adhesions and actin structures disassemble to make way for new, much more dynamic structures, thus enabling the subsequent front-rear polarization and eventual migration.

## Supplementary Materials

The following are available online at https://www.mdpi.com/2073-4409/9/3/578/s1, Figure S1: Immunostaining of p34 and E-cadherin in control and EGF-treated IAR-20 cells. Figure S2: Control IAR-20 cells expressing GFP-E-cadherin form a continuous monolayer at the beginning of the experiment. Figure S3: Immunofluorescent staining for β-catenin/actin/EPLIN in IAR-20 cells treated with EGF for 15 and 30 min. Figure S4: Western blot analysis of EPLIN. Video S1: Scattering of IAR-20 epithelial cells in response to EGF. Video S2: Disappearance of contact paralysis in the culture of IAR-20 epithelial cells in the presence of EGF. Video S3: Disruption of the circumferential actin bundle and appearance of pseudopodia at the cell-cell-boundaries after the addition of EGF. IAR-20 cells stably expressing F-tractin-tdTomato. Video S4: Myosin retrograde flow in IAR-20 cells treated with EGF. IAR-20 cells stably expressing GFP-RLC. Video S5: Displacement of ConA-coated beads attached to the dorsal cell surface in the zone of cell-cell contact before and after the addition of EGF. Video S6: Rearrangement of E-cadherin-based AJs in IAR-20 cells treated with EGF. IAR-20 cells stably expressing GFP-E-cadherin. Video S7: Rearrangement of E-cadherin-based AJs in IAR-20 cells treated with EGF. Enlarged region of Supplementary Video S6. Video S8: Accumulation of zyxin in AJs of IAR-20 cells treated with EGF. IAR-20 cells stably expressing GFP-E-cadherin were transfected with mKate2-zyxin.
