# Supplementary material for: Early Events in Actin Cytoskeleton Dynamics and E-Cadherin-Mediated Cell-Cell Adhesion during Epithelial-Mesenchymal Transition
Source: Cells. 2020 Feb 29;9(3):578. doi: 10.3390/cells9030578 (PMC7140442; doi:10.3390/cells9030578)
Supplement: Supplementary file 1 [file cells-09-00578-s001.zip › Supplementary_information-final.pdf]

SUPPLEMENTARY INFORMATION

Early events in actin cytoskeleton dynamics and E-cadherin-mediated cell-cell adhesion during epithelial-mesenchymal transition

Irina Y Zhitnyak[¶], Svetlana N Rubtsova[¶], Nikita I Litovka, Natalya A Gloushankova*

Institute of Carcinogenesis, N.N. Blokhin National Medical Research Center of Oncology, Moscow, Russia

* Corresponding author
E-mail: n.gloushankova@ronc.ru, natglu@hotmail.com
[¶] These authors contributed equally to this work.

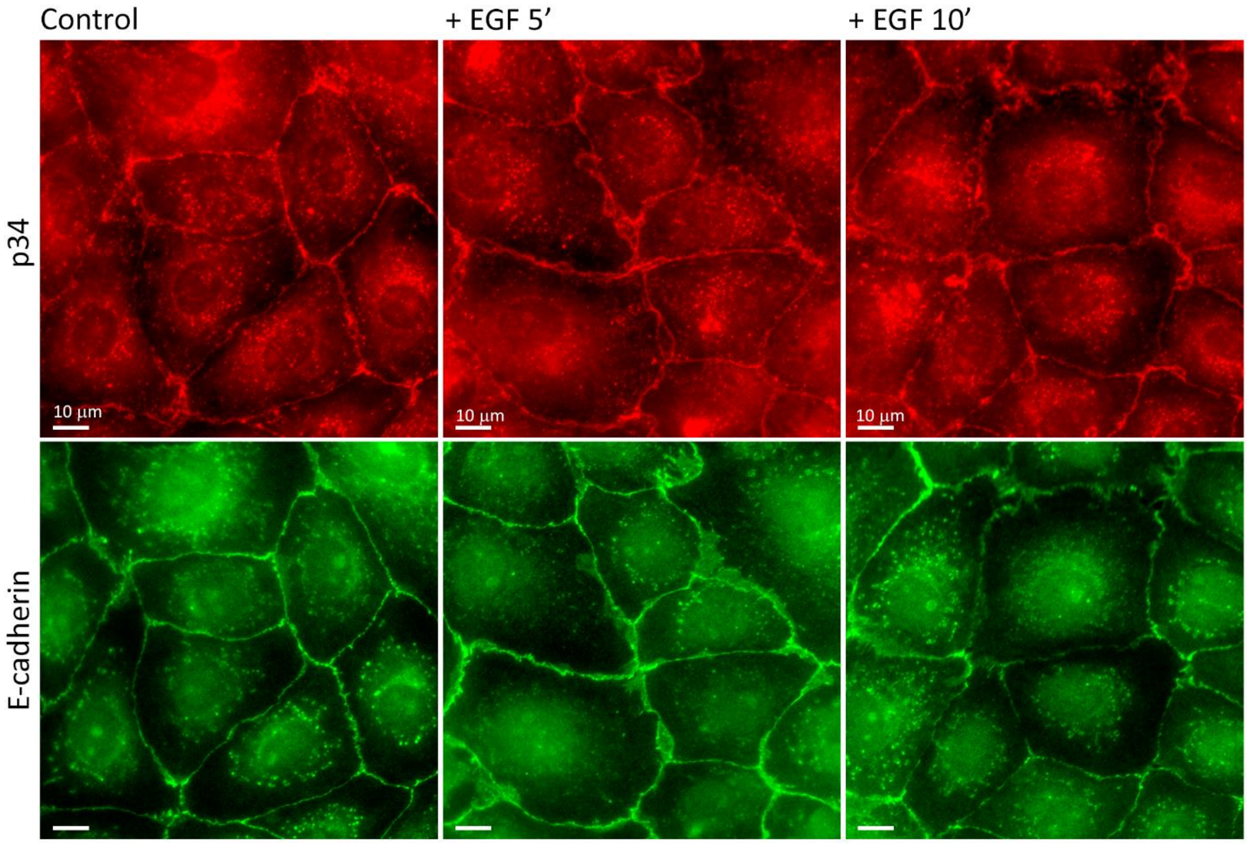

Supplementary Figure S1. Immunostaining of p34 and E-cadherin in control and EGF-treated IAR-20 cells.

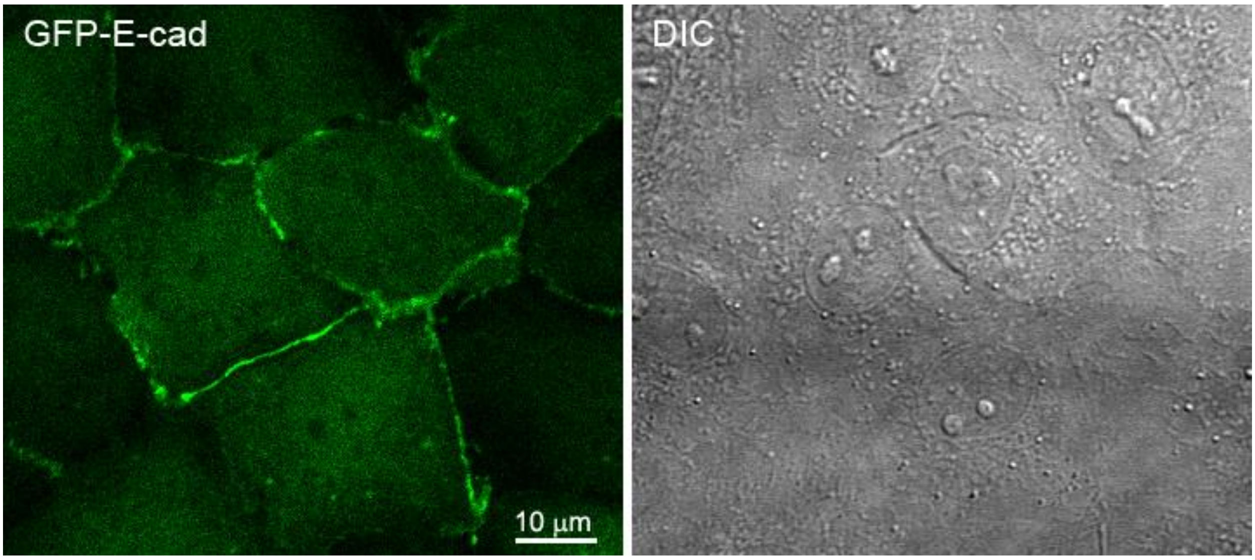

Supplementary Figure S2. Control IAR-20 cells expressing GFP-E-cadherin form a continuous monolayer at the beginning of the experiment.

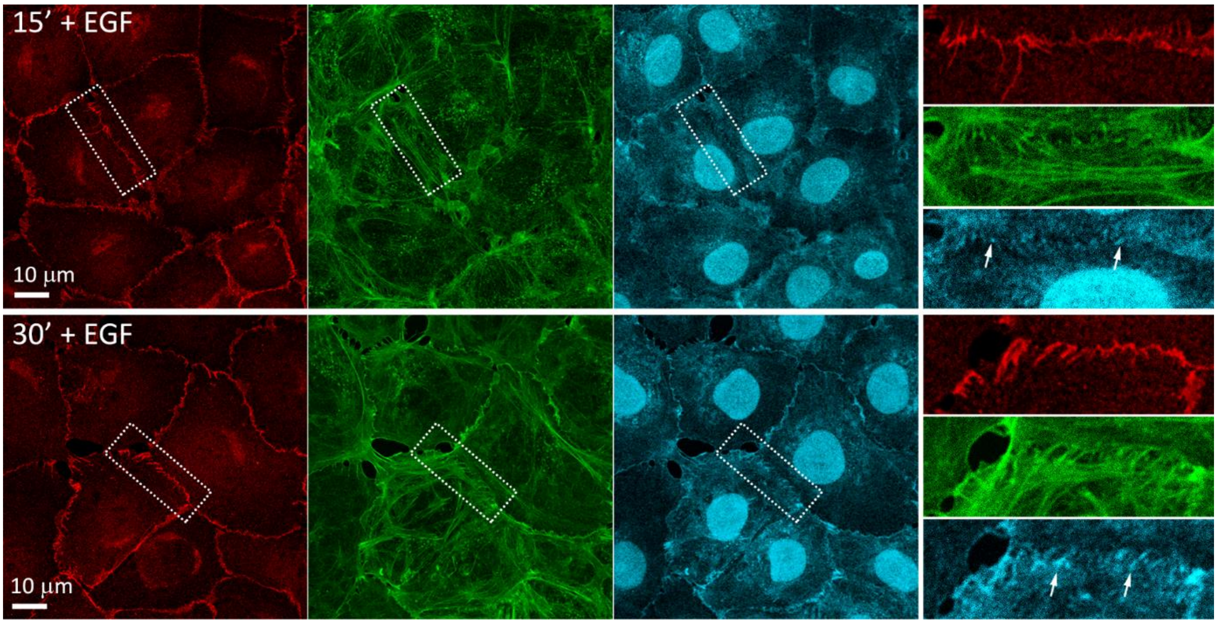

Supplementary Figure S3. Immunofluorescent staining for β-catenin/actin/EPLIN in IAR-20 cells treated with EGF for 15 and 30 min. Red – β-catenin, green – actin, blue – EPLIN. In cells treated with EGF (15 and 30 min) EPLIN is observed in straight actin fibers associated with punctate AJs (arrows). The enlarged cell-cell boundaries indicated with rectangles are shown on the right.

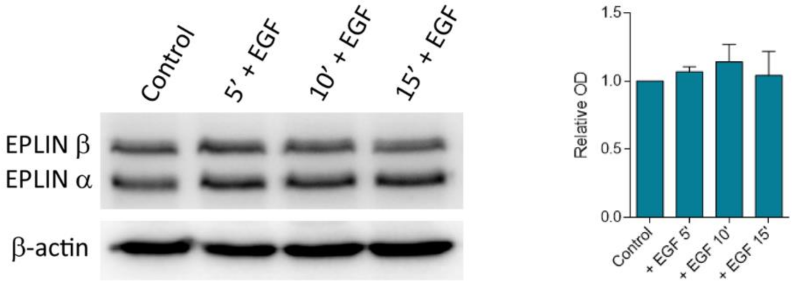

Supplementary Figure S4. Western blot analysis of EPLIN. Left – Western blot (10% PAAG) of EPLIN in the cells treated with EGF. β-actin was used as loading control. Right – western blot densitometry of EPLINβ relative to β-actin. Data are presented as mean ± SEM, N=3.

Legends to Supplementary Videos

Supplementary Video S1. Scattering of IAR-20 epithelial cells in response to EGF.

Supplementary Video S2. Disappearance of contact paralysis in the culture of IAR-20 epithelial cells in the presence of EGF.

Supplementary Video S3. Disruption of the circumferential actin bundle and appearance of pseudopodia at the cell-cell-boundaries after the addition of EGF. IAR-20 cells stably expressing F-tractin-tdTomato.

Supplementary Video S4. Myosin retrograde flow in IAR-20 cells treated with EGF. IAR-20 cells stably expressing GFP-RLC.

Supplementary Video S5. Displacement of ConA-coated beads attached to the dorsal cell surface in the zone of cell-cell contact before and after the addition of EGF.

Supplementary Video S6. Rearrangement of E-cadherin-based AJs in IAR-20 cells treated with EGF. IAR-20 cells stably expressing GFP-E-cadherin.

Supplementary Video S7. Rearrangement of E-cadherin-based AJs in IAR-20 cells treated with EGF. Enlarged region of Supplementary Video S6.

Supplementary Video S8. Accumulation of zyxin in AJs of IAR-20 cells treated with EGF. IAR-20 cells stably expressing GFP-E-cadherin were transfected with mKate2-zyxin.
